# Concurrent Denosumab and Parenteral Iron Therapy Precipitating Severe Hypocalcemia and Hypophosphatemia

**DOI:** 10.1210/jcemcr/luae005

**Published:** 2024-02-01

**Authors:** Sylvia Ye, Vivian Grill, Jinghang Luo, Hanh H Nguyen

**Affiliations:** Endocrinology and Diabetes Unit, Western Health, Melbourne, VIC 3021, Australia; Endocrinology and Diabetes Unit, Western Health, Melbourne, VIC 3021, Australia; Department of Medicine, The University of Melbourne, Melbourne, VIC 3021, Australia; Endocrinology and Diabetes Unit, Western Health, Melbourne, VIC 3021, Australia; Endocrinology and Diabetes Unit, Western Health, Melbourne, VIC 3021, Australia; Department of Medicine, The University of Melbourne, Melbourne, VIC 3021, Australia

**Keywords:** FGF23, iron infusion, denosumab, hypocalcemia, hypophosphatemia

## Abstract

Denosumab-induced hypocalcemia and iron infusion–related hypophosphatemia are both well described. We describe a case of severe hypocalcemia and hypophosphatemia following sequential denosumab and parenteral iron administration. This resulted in respiratory failure due to muscle weakness and cardiac arrhythmia, requiring noninvasive ventilation and urgent intravenous electrolyte replacement. This case highlights the severe dysregulation in calcium and phosphate homeostasis that can occur with denosumab and iron infusions when administered in quick succession. Given that these drugs are among the most common therapies prescribed across a range of specialties, we hope to alert clinicians to this potential serious drug-drug interaction and suggest strategies for monitoring and management of the electrolyte derangement.

## Introduction

Parenteral iron and denosumab are commonly prescribed in inpatient and community settings. Denosumab use has rapidly increased since its approval in 2010, and it is one of the most commonly prescribed osteoporosis therapies [1, 2]. Although fracture efficacy is well established, denosumab cessation has been associated with rebound bone loss and fractures [1], and denosumab-induced hypocalcemia can occur, particularly in advanced chronic kidney disease and vitamin D deficiency [3]. With increasing indications, parenteral iron is also increasingly prescribed by a range of clinicians, including primary care, gastroenterology, nephrology, hematology, and internal medicine physicians [4]. Parenteral iron impairs inactivation of fibroblast growth factor 23 (FGF23), leading to phosphaturia and reduced circulating 1,25-(OH)2 vitamin D, in turn reducing gastrointestinal phosphate absorption [5, 6]. FGF23-mediated hypophosphatemia occurs as a complication in up 45% to 47% of iron infusions, highest in those receiving ferric carboxymaltose (FCM) [7, 8].

Severe hypocalcemia and hypophosphatemia caused by iron infusion and denosumab administered in close proximity has been reported [9‐13]. Raising awareness of this potential interaction is critical to avoid life-threatening electrolyte derangement.

## Case Presentation

**Figure 1.**
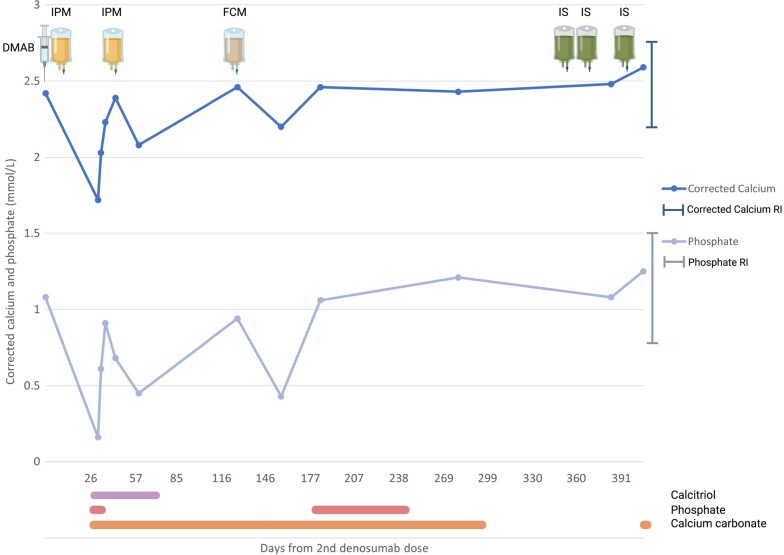
Timeline of serum albumin-corrected calcium and phosphate in relation to denosumab and iron infusions (iron polymaltose, ferric carboxymaltose, iron sucrose). Created with BioRender.com. Abbreviations: DMAB, denosumab; FCM, ferric carboxymaltose; IPM, iron polymaltose; IS, iron sucrose; RI, reference interval. ^a^Baseline levels of corrected calcium and phosphate were ten months prior to denosumab.

A 76-year-old man presented to the Emergency Department with lethargy, chest discomfort, and respiratory distress. Osteoporosis, based on a dual-energy x-ray absorptiometry (DXA)-derived bone density T-score at the hip and spine of −2.5 and −1.7 respectively, had been diagnosed by his primary care physician, who prescribed denosumab. There were no previous fractures. Kidney function, calcium, phosphate, and vitamin D levels had been normal. The first denosumab injection resulted in no known complications. There was also a history of iron deficiency anemia secondary to gastrointestinal angioectasias requiring 3 monthly iron infusions for the last year. Four weeks prior to presentation, the patient had received a second dose of subcutaneous denosumab 60 mg, and within 2 weeks was also administered intravenous iron polymaltose (IPM) (Fig. 1). He was also a heavy smoker with chronic obstructive airways disease, ischemic heart disease, cardiac failure, type 2 diabetes mellitus, hypertension, and a toxic multinodular goiter treated with antithyroid medications.

Two weeks after the iron infusion, he was hospitalized with severe hypophosphatemia and hypocalcemia, with serum phosphate < 0.16 mmol/L (< 0.50 mg/dL) (reference range [RR]: 0.75-1.50 mmol/L, 2.32-4.64 mg/dL), serum albumin-corrected calcium 1.72 mmol/L (6.88 mg/dL) (RR: 2.15-2.65 mmol/L, 8.60-10.60 mg/dL), serum albumin 34 g/L (3.4 g/dL) (RR: 34-47 g/L, 3.4-4.7 g/dL), and serum ionized calcium 0.92 mmol/L (3.68 mg/dL) (RR: 1.15-1.30 mmol/L, 4.60-5.20 mg/dL). The iron deficiency anemia had improved post-IPM, hemoglobin 103 g/L (10.3 g/dL) (RR: 130-180 g/L, 13-18 g/dL) and serum ferritin 149 ng/mL (149 μg/L) (RR: 30-320 ng/mL, 30-320 μg/L). There was a respiratory acidosis on venous gas and QT interval prolongation of 529 ms corrected for heart rate (QTc; normal 350-450 ms). He had nonsustained ventricular tachycardia while on cardiac telemetry. A normal serum creatinine 95 μmol/L (1.07 mg/dL) (RR: 60-110 μmol/L, 0.68-1.24 mg/dL), estimated glomerular filtration rate 67 mL/min/1.73 m^2^ (normal > 60), mild hypomagnesemia with serum magnesium 0.57 mmol/L (1.39 mg/dL) (RR: 0.60-1.10 mmol/L, 1.46-2.67 mg/dL), elevated serum parathyroid hormone (PTH) 36.9 pmol/L (348.0 pg/mL) (RR: 2.0-8.5 pmol/L, 18.9-80.2 pg/mL), and sufficient serum 25-OH vitamin D 67 nmol/L (26.8 ng/mL) (normal > 50 nmol/L, > 20 ng/mL) were found. His chest x-ray was clear and there was no troponin rise.

Respiratory support with noninvasive ventilation and urgent intravenous calcium gluconate and phosphate were administered. His respiratory acidosis resolved and QTc returned to the patient's baseline of 470 ms. Oral calcitriol, calcium carbonate, and phosphate supplementation were also commenced, and gradually weaned following discharge. Calcitriol was required for 4 weeks, and oral calcium and phosphate, with intermittently higher doses, were required for up to 12 months (Fig. 1).

## Diagnostic Assessment

Two further iron infusions with IPM and FCM within 3 months were required despite multiple argon plasma coagulation treatments of angioectatic lesions (Fig. 1). Each subsequent iron infusion resulted in transient hypophosphatemia, but without hypocalcemia. A nadir serum phosphate of 0.43 mmol/L (1.33 mg/dL) was documented after FCM. Phosphate levels normalized with oral phosphate and calcitriol.

The patient was referred to the Metabolic Bone Disorder Clinic for investigation. Four weeks following FCM, the fractional excretion of phosphate was elevated at 36% (10%-20%). FGF23 levels were not available at the time of hypophosphatemia but were subsequently normal 10 weeks after his last FCM. Serum 1,25-(OH)2 vitamin D measured after calcitriol commencement was 106 pmol/L (44.2 pg/mL) (RR: 50-190 pmol/L, 20.8-79.2 pg/mL). A secondary osteoporosis screen was negative.

## Treatment

A presumptive diagnosis of recurrent iron infusion–related, FGF23-mediated hypophosphatemia was made, with a serious denosumab and iron infusion interaction causing severe hypocalcemia and hypophosphatemia. Denosumab was ceased due to concern for iron infusion–related osteomalacia and risk of electrolyte disturbance, given the likely need for further iron infusions. This was considered to outweigh the risk of rebound bone loss and fractures following denosumab cessation, particularly in the context of short duration of denosumab therapy. Recommendations were made to consider alternative preparations of iron and avoid future administration of IPM and FCM.

Subsequent parenteral iron therapy was changed to iron sucrose, which has since been administered on multiple occasions with close monitoring, with no further hypophosphatemia and normal FGF23 levels.

## Outcome and Follow-Up

Serum bone turnover markers 5 and 9 months after his last dose of denosumab were suppressed, with C-terminal telopeptide of type 1 collagen 248 ng/L (RR: 100-750 ng/L) and procollagen-1 N-terminal peptide 33 μg/L (RR: 15-115 μg/L). A repeat DXA at 24 months has shown stable hip and spine bone density and there were no new vertebral fractures on Vertebral Fracture Analysis. The patient has not sustained any clinical fractures in 2.5 years of follow-up.

## Discussion

We present a case of severe symptomatic hypocalcemia and hypophosphatemia after co-administration of intravenous iron and denosumab, highlighting the dysregulation in calcium and phosphate homeostasis that can occur even in the setting of normal renal function and vitamin D levels. Given that these drugs are commonly prescribed across a range of specialties, and acute hypophosphatemia and hypocalcemia can cause tetany, cardiorespiratory failure, seizures, and coma in severe cases [5, 7, 8], clinicians need to be alert to this potential serious drug-drug interaction.

Since 2016, there have been 6 published cases of electrolyte derangement following co-administration of denosumab and parenteral iron [9‐13] (Table 1). Previously described cases were frequently asymptomatic and occurred in patients with varying renal function and 25-OH vitamin D levels. The present case was symptomatic and had complications of respiratory failure, QTc prolongation, and arrythmia, which resolved with urgent electrolyte replacement. In previously published cases [9‐13], electrolyte disturbance occurred at 8 to 28 days after denosumab and 8 days to 2 months after intravenous iron. Iron and denosumab were given on the same day to 16 days apart, with the exception of one patient on years of recurrent iron infusions where the drugs were 2 months apart [10]. Our patient similarly presented 4 weeks after denosumab and 2 weeks after IPM. It is likely that the risk is highest in the first month following denosumab administration.

**Table 1.** Summary of findings from previously published cases of hypophosphatemia and hypocalcemia with iron infusions and denosumab

Author, year	Patient background (age, sex, clinical details)	IV iron type, dose, timing*^a^* Denosumab dose, timing*^a^*	Symptoms, electrolyte nadir	Management and recovery
Smyth and Ong, 2016 (9)	59F CKD stage 3 (eGFR 31) Osteoporosis, unknown if clinical fractures	IPM (1st dose) 1 g 21 days prior Denosumab (2nd dose) 60 mg 26 days prior	Asymptomatic PO_4_ 0.36 mmol/L (1.11 mg/dL) (L) cCa 1.81 mmol/L (7.25 mg/dL) PTH 40.2 mmol/L (379.1 pg/dL) (H) 25-OH vitD 55 nmol/L (22 ng/mL) FePO_4_ 71.6% (H)	IV calcium and phosphate replacement Discharged after 3 days on calcitriol 0.5 μg twice daily, calcium carbonate 1.2 g daily, phosphate 500 mg daily, weaned over 3 months
Fang et al. 2019 (10)	73 F GI bleeding from portal hypertensive gastropathy with GAVE	FCM recurrent 1 g over 2 years, last dose 2 months prior Denosumab (1st dose) 1 month prior	10 months of proximal muscle pain and weakness, osteomalacia-related fractures PO_4_ 0.27 mmol/L (0.84 mg/dL) (L) cCa 2.04 mmol/L (8.16 mg/dL) (L) PTH 29.8 pmol/L (281.0pg/dL) (H) 25-OH vitD 32 nmol/L (12.8 ng/mL) (L)	Multiple IV phosphate infusions (150 mmol total) and oral replacement Phosphate recovery over 3 weeks Symptoms resolved and biochemistry normal at 6months
Cohen and Chacko, 2022 (11)	83 M CKD stage 4 (eGFR 19)	FCM 1 g 1 month prior Denosumab 60 mg 3 weeks prior	Unknown if symptomatic PO_4_ 0.57 mmol/L (1.76 mg/dL) (L) cCa 1.66 mmol/L (6.65 mg/dL) (L) iCa 0.89 mmol/L (3.92 mg/dL) (L) PTH 94.6 pmol/L (892.1 pg/dL) (H)	Not specified
Cohen and Chacko, 2022 (11)	76 M CKD stage 4 (eGFR 17) Osteoporosis	FCM within 1 month prior Denosumab within 1 month prior	Unknown if symptomatic PO_4_ 0.8 mmol/L (2.48 mg/dL) cCa 1.77 mmol/L (7.09 mg/dL) (L) iCa 1.00 mmol/L (4.00 mg/dL) (L) PTH 100 pmol/L (943 pg/dL) (H) 25-OH vitD normal	Not specified
Tai, et al. 2022 (12)	>75 F Post minimal trauma fracture of neck of femur Normal renal function (eGFR > 89)	FCM (1st dose) 1 g 8 days prior Denosumab (1st dose) 60 mg 8 days prior	Asymptomatic PO_4_ 0.37 mmol/L (1.15 mg/dL) (L) cCa 2.07 mmol/L (8.28 mg/dL) (L) iCa 1.07 mmol/L (4.28 mg/dL) (L) 25-OH vitD 51 nmol/L (20.4 ng/mL)	IV phosphate 30 mmol/L, oral phosphate replacement 1000 mg twice daily Calcitriol 0.5 μg daily, calcium carbonate 1200 mg 3 times daily Cholecalciferol increased from 25 μg to 50 μg daily Normalization at 12 days post co-administration
Muhandiramge, et al. 2023 (13)	92 F Post minimal trauma fracture of neck of femur and distal radius eGFR 63	FCM 27 days prior Denosumab (1st dose) 11 days prior	Asymptomatic PO_4_ < 0.30 mmol/L (< 0.93 mg/dL) (L) cCa 1.50 mmol/L (6.01 mg/dL) (L) 25-OH vitD 74 nmol/L (29.6 ng/mL)	Oral calcium replacement No phosphate replacement Calcium normalized at 46 days post denosumab, phosphate between 11-35 days post denosumab

Reference ranges: PO4 0.75-1.50 mmol/L (2.33-4.65 mg/dL), cCa 2.15-2.65 mmol/L (8.62-10.62 mg/dL), iCa 1.15-1.30 mmol/L (2.60-5.20 mg/dL), PTH 2.0-8.5 pmol/L (18.9-80.2 pg/mL), 25-OH vitD >50 nmol/L (> 20 ng/mL), FePO4 10-20%

Abbreviations: BMD, bone mineral density; cCa, corrected calcium; CKD, chronic kidney disease; eGFR, estimated glomerular filtration rate; FePO4, fractional excretion of phosphate; IPM, iron polymaltose; FCM, ferric carboxymaltose; GAVE, gastric antral vascular ectasia; GI, gastrointestinal; iCa, ionized calcium; IV, intravenous; PO_4,_ serum phosphate; PTH, parathyroid hormone; 25-OH vitD, 25-OH vitamin D.

^*a*^Prior to presentation or first electrolyte disturbance noted.

**Figure 2.**
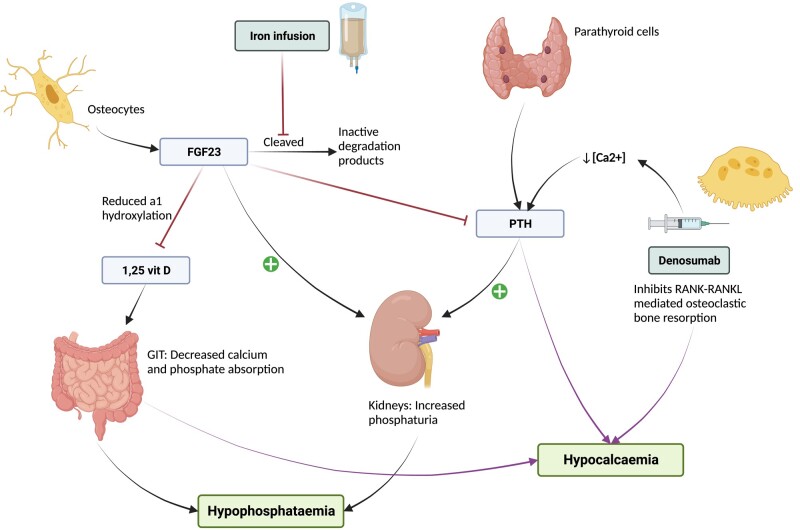
Proposed mechanism of hypocalcemia and hypophosphatemia with denosumab and iron infusions in our patient. Created with BioRender.com. Abbreviations: FGF23, fibroblast growth factor 23; GIT, gastrointestinal tract; PTH, parathyroid hormone; RANK/L, receptor activator of NF-κB/ligand.

The electrolyte disturbance which has been observed in patients receiving intravenous iron and denosumab is due to alterations in PTH, 1,25-(OH)2 vitamin D, and FGF23 (Fig. 2). Denosumab inhibits osteoclastic bone resorption, transiently reducing plasma calcium concentration, and resulting in elevated PTH levels [5]. This secondary hyperparathyroidism decreases renal phosphate reabsorption, promoting phosphaturia. Iron infusions inhibit cleavage and inactivation of FGF23 [6]. High levels of FGF23 decrease renal phosphate reabsorption and inhibit renal 1α-hydroxylation of 25-OH vitamin D to activated 1,25-(OH)2 vitamin D, thereby impairing intestinal absorption of calcium and phosphate [5]. Since FGF23 impairs 1,25-(OH)2 vitamin D production, the compensatory response to hypocalcemia induced by denosumab is blunted in patients also receiving an iron infusion. Hence co-administration of iron infusions and denosumab are expected to amplify both the hypocalcemia and hypophosphatemia.

We would have expected our patient to have elevated FGF23, low 1,25-(OH)2 vitamin D, and high fractional excretion of phosphate during his time of hypophosphatemia and hypocalcemia, but they were not measured at that time. Normal FGF23 levels 10 weeks after intravenous FCM were found, at a time of normal serum phosphate. His 1,25-(OH)2 vitamin D levels were also only checked after oral calcitriol. Hypophosphatemia and elevated FGF23 were not observed after changing to iron sucrose. This may be due to the waning effect of denosumab on PTH, or it may support the proposal that different iron preparations have varying risk of hypophosphatemia [7, 8]. Meta-analyses of randomized controlled trials, FCM was associated with an almost 8-fold and 9-fold higher risk of hypophosphatemia compared with ferric derisomaltose (FDI) and iron sucrose, respectively [8]. Therefore, in patients requiring ongoing iron infusions, consideration should be given to trialing formulations less likely to cause hypophosphatemia.

We were concerned about osteomalacia in our patient, given that recurrent iron infusion–related hypophosphatemia has been associated with osteomalacia and increased fracture risk [7, 8, 14]. In a similar case described by Fang et al [10], a 73-year-old woman who had received 2 years of recurrent iron infusions presented with bone pain, hypophosphatemia 0.27 mmol/L (0.84 mg/dL), and hypocalcemia 2.04 mmol/L (8.18 mg/dL) following her first dose of denosumab, and she was subsequently found to have osteomalacia-related fractures. In our patient, denosumab was ceased as a precaution. No transition to bisphosphonate therapy was planned, as it is contraindicated in iron infusion–related osteomalacia[14], and risk of rebound bone loss was thought to be lower following 2 doses of denosumab. Reassuringly, no evidence of increased bone turnover, loss of bone density, or clinical fractures have been observed over 2.5 years of follow-up.

**Figure 3.**
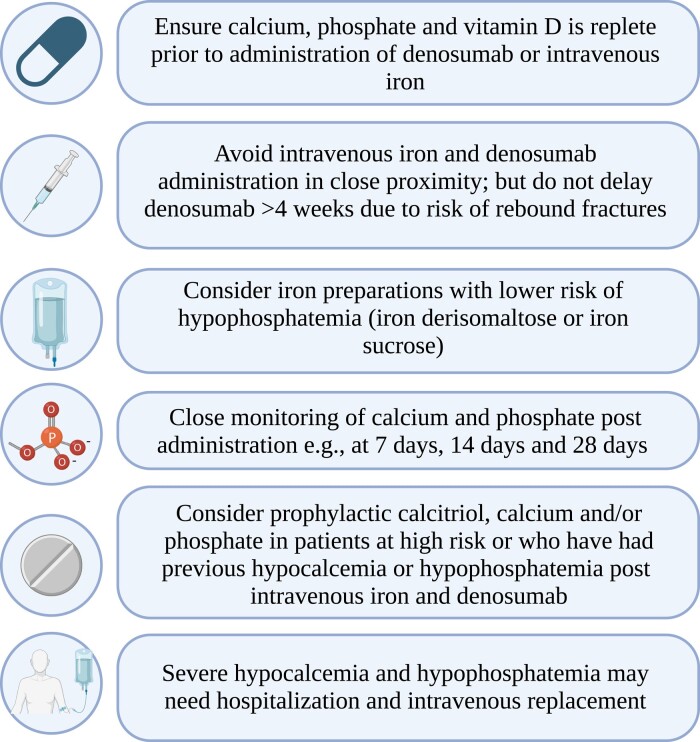
Reducing risk of hypocalcemia and hypophosphatemia: considerations in patients requiring intravenous iron and denosumab. Created with BioRender.com

Currently, there is lack of guidance on managing patients on denosumab therapy who also require iron infusions. This poses a management dilemma as long-term denosumab users cannot have their therapy delayed due to the risk of rebound bone loss and fractures. We recommend close monitoring of serum calcium and phosphate at 7, 14, and 28 days if co-administration cannot be avoided (Fig. 3). Based on our case and that of previously described cases [9‐13], the highest risk period appears to be in the first month following denosumab administration, and cautious deferral of denosumab therapy for < 4 weeks could be considered. Evidence of hypocalcemia or hypophosphatemia should be promptly treated with calcitriol (given FGF23-mediated impairment of 1,25-(OH)2 vitamin D activation), as well as oral and/or intravenous calcium and phosphate replacement. Prophylactic replacement may be needed if ongoing intravenous iron and denosumab therapy is required. As hypomagnesemia can impair PTH secretion and activation of PTH receptors [15], correction of hypomagnesemia is also important. Given the lower reported risk with iron sucrose and FDI, use of these agents over FCM and IPM could be considered in those treated with denosumab, although further research is required to confirm in this population.

In summary, this case highlights that co-administration of iron infusions and denosumab may lead to severe life-threatening electrolyte derangement and awareness of the interaction will hopefully lead to strategies to monitor and mitigate the electrolyte abnormalities. Further prospective studies are required to determine the frequency of this serious but likely underdiagnosed drug-drug interaction, the timing of electrolyte disturbance in order to refine monitoring strategies, and whether different iron preparations reliably reduce the risk of hypophosphatemia.

## Learning Points

- Although denosumab-induced hypocalcemia and iron infusion–related hypophosphatemia are well described, co-administration of these agents may lead to severe life-threatening electrolyte derangement requiring hospital admission and intravenous replacement.
- Awareness of a drug interaction between denosumab and iron infusions should lead to strategies to monitor and mitigate the electrolyte abnormalities, that is, avoiding co-administration without delaying denosumab > 4 weeks, using alternative intravenous iron preparations associate with lower risk, and close monitoring of serum calcium and phosphate up to 1 month after administration.
- Further research is necessary to determine the frequency and timing of this drug interaction, and to assess whether the use of alternative iron preparations may reliably reduce risk of developing an interaction.

## Contributors

All authors made individual contributions to authorship. S.Y. was involved in management of this patient and primary manuscript draft and submission. V.G., J.L., and H.H.N. were involved in diagnosis and management of this patient and manuscript revision. All authors reviewed and approved the final draft.

## Data Availability

Data sharing is not applicable to this article as no datasets were generated or analyzed during the current study.

## Abbreviations

DXA: dual-energy x-ray absorptiometry
FCM: ferric carboxymaltose
FDI: ferric derisomaltose
FGF23: fibroblast growth factor 23
IPM: intravenous iron polymaltose
PTH: parathyroid hormone
RR: reference range
